# Differential expression of papillomavirus L1 proteins encoded by authentic and codon modified L1 genes in methylcellulose-treated mouse keratinocytes

**DOI:** 10.1186/1743-422X-4-127

**Published:** 2007-11-25

**Authors:** Xiao Wang, Bo Li, Kong-Nan Zhao

**Affiliations:** 1 Diamantina Institute for Cancer, Immunology & Metabolic Medicine, University of Queensland, Research Extension, Building 1, Princess Alexandra Hospital, Woolloongabba, Queensland 4102, Australia

## Abstract

Papillomaviruses (PVs) are double-stranded DNA viruses that infect keratinocytes in differentiating epithelia and induce hyperproliferative lesions. Here, we used methylcellulose to induce cell differentiation of primary mouse keratinocytes (KCs) in *in vitro *culture and assessed the expression of authentic and codon-modified version of L1 capsid genes from two PV types (HPV6b and BPV1). Based on the quantitative RT-PCR analysis, methylcellulose treatment did not influence the transcriptional expression of both authentic and codon-modified L1 genes in KCs. Western blot showed that methylcellulose significantly increased the levels of the L1 proteins expressed from two authentic L1 genes. Conversely, methylcellulose dramatically decreased L1 protein expression in KCs transfected with two codon-modified L1 expression constructs. These data suggest that L1 protein expression is associated with KC differentiation induced by methylcellulose treatment and regulated at the post-transcriptional level.

## Findings

Papillomaviruses (PVs) are double-stranded DNA viruses that infect keratinocytes in differentiating epithelia and induce hyperproliferative lesions [1]. Amplification of PV DNA and transcription of PV late genes is activated in suprabasal cells of differentiated epithelium, indicating that the PV life cycle is closely linked to host cell differentiation [2]. This link has posed a substantial barrier to the study of PV in the laboratory because PVs cannot be propagated in conventional cell lines. Different raft culture systems that mimick keratinocyte differentiation *in vitro *have been developed to study viral gene transcription [3] and to achieve differentiation-specific viral amplification and virion morphogenic stages [4] and to produce virions from infected cells for sexually transmitted HPV types [5,6]. However, the yield of infectious virus is very low in those systems. Because raft culture is a time-consuming technique, it cannot be used for rapid analysis of multiple constructs [7].

Recently, we established mouse primary KCs culture system to express PV L1 proteins by transient transfection of authentic or codon modified L1 gene expression constructs [8]. Using the KC culture system, we proved that KC differentiation differentially regulates expression of PV authentic and codon modified L1 genes [8,9]. Methylcellulose is a cell differentiation enhancer widely used in the study of KC differentiation [10-12]. Human KCs grown in methylcellulose semisolid medium for 48 h were induced to differentiate and express involucrin a terminal KC differentiation marker [13,14]. In HPVs, Flores and Lambert reported that HPV 16 DNA replication was promoted and virus-like particles were detected when HPV-16-positive cervical epithelial cells were grown in medium containing 1.68% methylcellulose for 2 to 10 days [15]. Methylcellulose also induced HPV31-positive epithelial cells to express two KC terminal differentiation markers involucrin and transglutaminase [16]. However, no HPV 31 L1 protein expression was detected in HPV-infected KCs treated by methylcellulose although L1 mRNA was well transcribed [16]. In this work, we investigated effects of methylcellulose treatment on expression of PV L1 genes in our established mouse primary KC culture system. Four PV L1 gene expression constructs including two authentic (*Nat*) L1 gene plasmids (pcDNA3HPV6b *Nat *L1 and pcDNA3BPV1 *Nat *L1) and two codon modified (*Mod*) L1 gene plasmids (pcDNA3HPV6b *Mod *L1, and pcDNA3BPV1 *Mod *L1) were used in the experiments as previously described [8].

**Figure 1:**
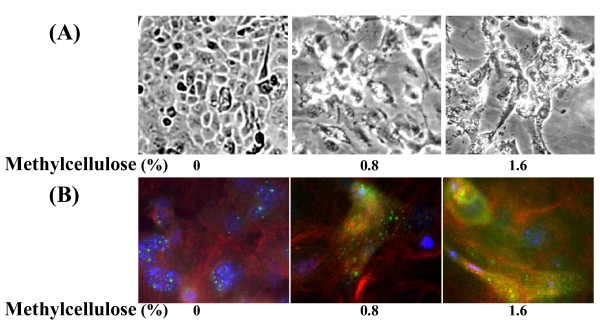
**Cell morphology and expression of involucrin in the primary mouse KCs grown in KC-SF medium containing different concentration of methylcellulose for 2 days**. **(A)**. Gross cell morphology. Images ×200.**(B)**. Immunofluorescence micrograph showing involucrin (green), β-tubulin (red) and nuclei (blue). Images ×400.

We prepared primary KCs from new-born mouse skin as previously described [8]. The primary mouse KCs were directly grown in semisolid KC-SF complete medium (Gibco, Australia) containing 0, 0.8% and 1.6% methylcellulose for two days. Resulting morphological changes of the cultured KCs after methylcellulose treatment were clearly observed, which included the changes of cell sizes and shapes (Fig 1A). The morphological changes of the cultured KCs were tightly associated with methylcellulose concentrations. The KCs changed their morphologies more dramatically in medium containing 1.6% methylcellulose than in medium containing 0.8% methylcellulose (Fig 1A). Immunofluorescence microscopy revealed further that the KCs grown in methylcellulose-free medium for two days showed weak involucrin signals, but expression of involucrin was significantly up-regulated in the KCs grown in medium containing methylcellulose (Fig. 1B). Methylcellulose treatment also resulted the KCs to change expression patterns of the other KC differentiation markers by reducing expression of basal keratins K14 and increasing expression of keratins K1 and K10 (data not shown). These data indicate that methylcellulose could induce mouse primary KCs to rapidly differentiate, consistent with previous observations for human KCs [13,14].

**Figure 2:**
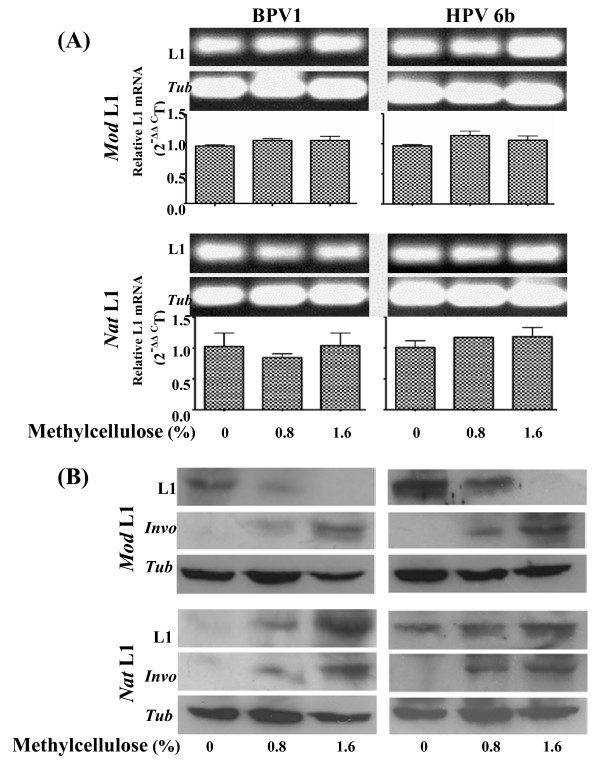
**Effects of post-transfection treatment of methylcellulose on expression of PV L1 genes in mouse primary KCs**. Newly isolated mouse primary KCs, respectively transfected with four PV L1 expression constructs, were grown in basal KC medium for 18 h and in 3:1 medium for 24 h. The L1-tranfected KCs were suspended in semisolid KC medium containing different concentration of methylcellulose for 48 h and harvested for analysis of L1 gene expression. **(A)**. L1 transcripts were assessed by quantitative RT-PCR. β-tubulin transcript was analysed as an internal control. *Up panel: *Representative electrophoresis of the L1 and tubulin mRNA qRT-PCR products. *Lower panel*: Results are shown with the means (± S.E.M) of duplicate transfection assays from two separate experiments. **(B)**. Expression of the L1 proteins analysed by Western blot is representative of duplicate transfection from two separate experiments. β-tubulin (*Tub*) was used as comparable loading control.

We first examined whether and how post-transfection treatment of methylcellulose affected expression of the four PV L1 expression constructs in the transiently L1-transfected KCs. Separated batches of 1 × 10^6 ^cells of the freshly isolated mouse primary KCs were respectively transfected with 2 μg of each of the four PV L1 plasmid DNAs using lipofectamine (Invitrogen, Australia). The L1-transfected KCs were incubated in basal KC medium for 18 h, and then grown in semisolid KC-SF complete medium containing different concentrations of methylcellulose (0%, 0.8% and 1.6%) for 48 h. The L1-transfected KCs treated with methylcellulose were harvested for analysing L1 gene expression at both transcriptional and translational levels (Fig 2). Total mRNAs were extracted from the L1-transfected KCs and reverse-transcribed into cDNA using a reverse transcription kit (Promega, Australia). The cDNAs were analyzed by quantitative RT-PCR for the L1 and tubulin transcripts using SYBR^® ^Greet PCR kit (Qiagen, Australia). The specificity of RT-PCR was determined by the Rotor Gene Software and agarose gel electrophoresis (Fig 2A). As shown in Fig 2A, L1 transcripts were present at a similar level in L1-transfected KCs treated with 0%, 0.8% and 1.6% methylcellulose. These data indicate that L1 transcription was unrelated to methylcellulose treatment. Western blot analysis was used to measure L1 protein expression. Protein (40 μg) extracted from the L1-transfected KCs with or without methylcellulose treatment was separated by SDS-PAGE and blotted onto PVDF membrane. The blots were probed with either anti-HPV L1 monoclonal antibody (BD PharMingen, Australia) or anti-involucrin polyclonal antibody (Covance, USA) at 4°C over night. Blots were then incubated with horseradish-peroxidase(HRP)-conjugated goat anti-mouse IgG or HRP- conjugated goat anti-rabbit IgG (Sigma, Australia) followed by a chemiluminescence analysis (ECL, Amersham, Australia). Western blots showed that significantly up-regulated expression of involucrin was associated with the methylcellulose treatment (Fig 2B), indicating that methylcellulose enhanced L1-transfected KC differentiation. In the absence of methylcellulose treatment, only weak signals of the L1 proteins were detected in KCs transfected with the two PV *Nat *L1 expression constructs, but the levels of L1 proteins were significantly higher in KCs transfected with the two PV *Mod *L1 expression constructs (Fig 2B). Post-transfection treatment of methylcellulose dramatically decreased L1 protein expression in KCs transfected with the *Mod *PV L1 constructs (Fig 2B). Conversely, methylcellulose significantly increased the levels of the L1 proteins encoded by the two PV *Nat *L1 genes (Fig 2B). The down- and up-regulated responses of the L1 protein expression from the PV *Mod *and *Nat *L1 genes to post-transfection treatment of methylcellulose were clearly dose-dependent and associated with the differentiation status of the L1-tranfected KCs. The results support our previous study that gene codon composition in part determined differentiation-dependent expression of the PV L1 proteins in KCs [8].

**Figure 3:**
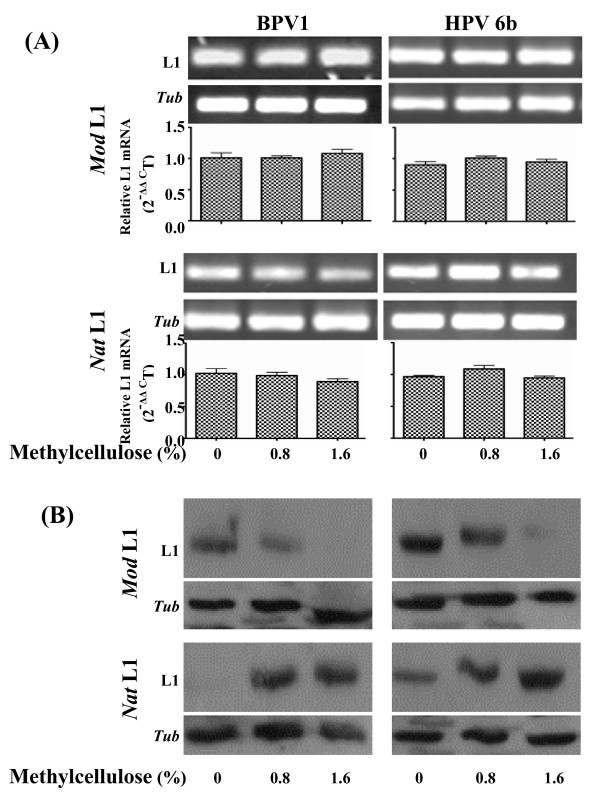
**Effects of pre-transfection treatment of methylcellulose on expression of the PV L1 genes in mouse primary KCs**. Newly isolated mouse KCs were suspended in semisolid KC medium containing different concentration of methylcellulose for 48 h. They were then transfected with the four PV L1 gene expression constructs and grown in KC-SF complete medium for 48 h before harvested for analysis of L1 gene expression. **(A)**. L1 transcripts were assessed by quantitative RT-PCR. β-tubulin transcript was analysed as an internal control. *Up panel: *Representative electrophoresis of the L1 and tubulin mRNA qRT-PCR products. *Lower panel*: Results are shown with the means (± S.E.M) of duplicate transfection assays from two separate experiments. **(B)**. Expression of the L1 proteins analysed by Western blot is representative of duplicate transfection from two separate experiments. β-tubulin (*Tub*) was used as comparable loading control.

We next examined how pre-transfection treatment of methylcellulose affected transcriptional and translational expressions of the four PV L1 expression constructs in transiently L1-transfected KCs. Freshly isolated primary mouse KCs were grown in semisolid KC-SF complete medium containing 0, 0.8% and 1.6% methylcellulose for two days. The KCs were recovered from semisolid KC-SF complete medium by multiple dilutions with serum-free F medium and PBS followed by centrifugation. The methylcellulose-treated KCs after recovered were respectively transfected with each of the four PV L1 plasmid DNAs using lipofectamine as mentioned above. At 48 h post-transfection, the L1-transfected KCs were harvested for analysing L1 transcripts and proteins (Fig. 3). Again, quantitative RT-PCR was used to examine transcripts of both *Nat *and *Mod *PV L1 genes, with no remarkable differences observed (Fig. 3A). Meantime, significantly up-regulated expression of involucrin was detected by Western blot analysis (data not shown). Western blot showed further that methylcellulose-induced KC differentiation resulted in dramatically down-regulated expression of the L1 proteins from the two PV *Mod *L1 genes (Fig. 3B), but significantly up-regulated expression of the L1 proteins from the two PV *Nat *L1 genes (Fig. 3B). These data indicate that pre-transfection treatment of methylcellulose also differentially regulated expression of the L1 proteins from PV *Nat *or *Mod *L1 genes, confirming further that expression of the L1 proteins from PV *Nat *and *Mod *L1 genes is differentially associated with the differentiation status of the KCs.

The close association of the HPV life cycle with the differentiation state of its host cell is demonstrated by the restriction of late gene transcription and amplification of viral DNA to suprabasal epithelial cells. The study of HPVs in cell culture has been hindered because of the difficulty in recreating the three-dimensional structure of the epithelium on which the virus depends to complete its life cycle. Although raft culture system can provide a spatial separation of cells for the study of HPV life cycle [3], it is technically challenging and requires extended periods of time for KC growth and differentiation. Meantime, it is hard to isolate separate layers in raft culture system. We developed the simple mouse primary KCs culture system to successfully express PV L1 proteins by transient transfection of the L1 expression constructs [8]. We reported that primary KCs in culture undergo cell differentiation to regulate expression of the PV L1 genes. Here, we demonstrated that suspension of mouse primary KCs in methylcellulose resulted in the rapid cell differentiation. As a model inducer of KC differentiation, use of methylcellulose has allowed us to characterize expression of targeted genes including L1 and involucrin in only 2–3 days instead of the 2 weeks required for raft culture and to study the mechanisms which regulate differentiation-dependent expression of the PV late genes. Methylcellulose did not influence L1 mRNA transcription in L1-transfected KCs, thus, the results confirmed that the expression of the L1 protein was post-transcriptionally regulated, consistent with previous studies [4,17]. Our results demonstrated further that the L1 gene codon composition correlated with the differentiation-dependent expression of the L1 protein in L1-transfected KCs grown in KC-SF complete medium with or without methylcellulose. This correlation can be well explained by our previous observations that composition of aminoacyl-tRNA pool changes during cell differentiation, which differentially favors translation of PV authentic and codon-modified L1 genes [18].

In conclusion, we established a methylcellulose culture system in the mouse primary KCs and demonstrated that methylcellulose enhanced KC differentiation. Methylcellulose did not influence L1 transcription but differentially regulated translation of the authentic and codon-modified L1 genes. These data support our previous study that L1 expression in response to differentiation is regulated at the post-transcriptional level.

## Abbreviations

KCs : keratinocytes;

PVs : Papillomaviruses.

## Competing interests

The author(s) declare that they have no competing interests.

## Authors' contributions

XW and BL conducted experiments together. XW wrote the manuscript. KNZ designed and coordinated the research efforts and edited the manuscript. All co-authors read and approved the final manuscript.

## References

[B1] Spink KM, Laimins LA (2005). Induction of the human papillomavirus type 31 late promoter requires differentiation but not DNA amplification. J Virol.

[B2] Doorbar J (2005). The papillomavirus life cycle. J Clin Virol.

[B3] McCance DJ, Kopan R, Fuchs E, Laimins LA (1988). Human papillomavirus type 16 alters human epithelial cell differentiation in vitro. Proc Natl Acad Sci U S A.

[B4] Frattini MG, Lim HB, Laimins LA (1996). In vitro synthesis of oncogenic human papillomaviruses requires episomal genomes for differentiation-dependent late expression. Proc Natl Acad Sci U S A.

[B5] Dollard SC, Wilson JL, Demeter LM, Bonnez W, Reichman RC, Broker TR, Chow LT (1992). Production of human papillomavirus and modulation of the infectious program in epithelial raft cultures. OFF. Genes Dev.

[B6] Meyers C, Frattini MG, Hudson JB, Laimins LA (1992). Biosynthesis of human papillomavirus from a continuous cell line upon epithelial differentiation. Science.

[B7] McLaughlin-Drubin ME, Christensen ND, Meyers C (2004). Propagation, infection, and neutralization of authentic HPV16 virus. Virology.

[B8] Zhao KN, Gu W, Fang NX, Saunders NA, Frazer IH (2005). Gene codon composition determines differentiation-dependent expression of a viral capsid gene in keratinocytes in vitro and in vivo. Mol Cell Biol.

[B9] Fang NX, Gu W, Ding J, Saunders NA, Frazer IH, Zhao KN (2007). Calcium enhances mouse keratinocyte differentiation in vitro to differentially regulate expression of papillomavirus authentic and codon modified L1 genes. Virology.

[B10] Watt FM, Jones PH (1993). Expression and function of the keratinocyte integrins. Dev Suppl.

[B11] Kubler MD, Jordan PW, O'Neill CH, Watt FM (1991). Changes in the abundance and distribution of actin and associated proteins during terminal differentiation of human epidermal keratinocytes. J Cell Sci.

[B12] Zhu S, Oh HS, Shim M, Sterneck E, Johnson PF, Smart RC (1999). C/EBPbeta modulates the early events of keratinocyte differentiation involving growth arrest and keratin 1 and keratin 10 expression. Mol Cell Biol.

[B13] Green H (1977). Terminal differentiation of cultured human epidermal cells. Cell.

[B14] Adams JC, Watt FM (1989). Fibronectin inhibits the terminal differentiation of human keratinocytes. Nature.

[B15] Flores ER, Lambert PF (1997). Evidence for a switch in the mode of human papillomavirus type 16 DNA replication during the viral life cycle. J Virol.

[B16] Ruesch MN, Stubenrauch F, Laimins LA (1998). Activation of papillomavirus late gene transcription and genome amplification upon differentiation in semisolid medium is coincident with expression of involucrin and transglutaminase but not keratin-10. J Virol.

[B17] Cumming SA, Repellin CE, McPhillips M, Radford JC, Clements JB, Graham SV (2002). The human papillomavirus type 31 late 3' untranslated region contains a complex bipartite negative regulatory element. J Virol.

[B18] Gu W, Ding J, Wang X, de Kluyver RL, Saunders NA, Frazer IH, Zhao KN (2007). Generalized substitution of isoencoding codons shortens the duration of papillomavirus L1 protein expression in transiently gene-transfected keratinocytes due to cell differentiation. Nucleic Acids Res.

